# RGB Colour Encoding Improvement for Three-Dimensional Shapes and Displacement Measurement Using the Integration of Fringe Projection and Digital Image Correlation

**DOI:** 10.3390/s18093130

**Published:** 2018-09-17

**Authors:** Luis Felipe-Sesé, Ángel Jesús Molina-Viedma, Elías López-Alba, Francisco A. Díaz

**Affiliations:** 1 Department of Mechanical Engineering, Campus Científico Tecnológico de Linares, University of Jaén, 23700 Linares, Spain; 2 Department of Mechanical and Mining Engineering, Campus Las Lagunillas, University of Jaén, 23071 Jaén, Spain; ajmolina@ujaen.es (A.J.M.-V.); elalba@ujaen.es (E.L.-A.); fdiaz@ujaen.es (F.A.D.)

**Keywords:** 3D shape, 3D displacements, fringe projection, digital image correlation, experimental mechanics

## Abstract

Three-dimensional digital image correlation (3D-DIC) has become the most popular full-field optical technique for measuring 3D shapes and displacements in experimental mechanics. The integration of fringe projection (FP) and two-dimensional digital image correlation (FP + DIC) has been recently established as an intelligent low-cost alternative to 3D-DIC, overcoming the drawbacks of a stereoscopic system. Its experimentation is based on the colour encoding of the characterized fringe and speckle patterns required for FP and DIC implementation, respectively. In the present work, innovations in experimentation using FP + DIC for more accurate results are presented. Specifically, they are based on the improvement of the colour pattern encoding. To achieve this, in this work, a multisensor camera and/or laser structural illumination were employed. Both alternatives are analysed and evaluated. Results show that improvements both in three-dimensional and in-plane displacement are obtained with the proposed alternatives. Nonetheless, multisensor high-speed cameras are uncommon, and laser structural illumination is established as an important improvement when low uncertainty is required for 2D-displacement measurement. Hence, the uncertainty has been demonstrated to be reduced by up to 50% compared with results obtained in previous experimental approaches of FP + DIC.

## 1. Introduction

Nowadays, full-field measurements of shape and displacements occurring on surface elements are accessible thanks to optical techniques based on digital cameras [1,2]. These techniques have provided interesting applications in experimental mechanics to observe and explain the mechanical behaviour of solids and the validation of theoretical and/or numerical models.

Although the theoretical basis of most optical techniques was established some decades ago, optical techniques are still improving their performance and capabilities due to technological advances, i.e., high-speed cameras, higher computational performance and improved data processing algorithms.

Several optical techniques are employed in experimental mechanics, depending on the mechanical variables of interest. For instance, digital photoelasticity [3,4,5], shows a high level of sensitivity in the direct measurement of the shear strain in photoelastic materials. Besides this, electronic speckle photography and interferometry techniques [2,6] offer high sensitivity for detecting displacements in the order of magnitude of fractions of the wavelength of the employed laser. In addition, thermoelasticity [2] allows us to experimentally obtain the variation of the first stress invariant from the thermal emissivity of a specimen under cyclic loading.

Other techniques are specifically focused on the measurement of shape and three-dimensional displacement, even in industrial environments, and are applicable for dynamic applications. Fringe projection (FP) [7,8,9,10,11] is a technique based on the Moiré effect which measures 3D shape, from which out-of-plane displacements can be extracted. It takes advantage of a fringe pattern (usually black and white) obliquely projected over the specimen whose image is recorded with a single camera. It is used in a wide range of applications, allowing sensitivities from microns to metres, depending on the optical set-up [11,12,13,14,15]. A more recent technique is digital image correlation 3D (3D-DIC) [16] which measures 3D shapes and displacements, from which strain fields can be calculated. This technique relies on the stereo-positioning and stereo-tracking of small facets of the image over time. An aleatory speckle pattern on the specimen surface is required (black dots randomly distributed on white background). This technique has multiple applications in experimental mechanics, either for static or high speed testing [17,18,19,20,21,22,23,24]. Its sensitivity strongly depends on the resolution of the camera and the optical arrangement, resulting in a range from microns to centimetres. Additionally, some work has been performed on digital image correlation with projected speckle patterns [25,26], but two cameras are still required.

Cameras employed in 3D-DIC technique require a perfect synchronisation (especially important for dynamic applications) and a complete calibration of the optical set-up to determine their relative orientation and the internal parameters of the cameras (i.e., extrinsic and intrinsic parameters) [16]. Computational and algorithm requirements usually make 3D-DIC difficult to implement, and the use of commercial software is often required. Additionally, the set-up significantly increases its cost when high-speed cameras are required for dynamic events. Moreover, a single camera is only required for the 2D version of DIC (2D-DIC) [27,28,29,30] but it is limited to in-plane displacement (Δ*x* and Δ*y*) measurement on flat specimens. Therefore, 2D-DIC and FP require lower equipment costs (only one camera and an additional projector), but they present some independent restrictions compared to 3D-DIC. However, these are complementary and could be overcome by their integration.

In fact, an alternative technique to 3D-DIC combining 2D-DIC and FP was investigated by previous researchers [31,32,33,34,35,36,37,38], but they were usually limited to static testing, a specific size of specimens or presented some errors in the measurement. Nonetheless, the authors of this work proposed a new integration methodology to measure large 3D shapes and displacements in solids [39,40]. This improved methodology overcame the independent restrictions presented by 2D-DIC and FP. Therefore, nowadays, it is possible to measure simultaneously in-plane (Δ*x*, Δ*y* with 2D-DIC) and out-of-plane displacements or shapes (Δ*z* with FP) using only one single camera and an additional fringe projector.

One of the most important issues in this integration is the distortion of in-plane displacements due to 3D surfaces or out-of-plane displacements. This aspect was neglected by some previous authors, and others tried to minimize it by employing telecentric lenses [31]. The authors of the current work developed a more practical optical correction which also resulted into a more economical and user-friendly solution and a feasible alternative method to 3D-DIC [39]. The developed method employs a CCD camera with conventional lenses and a post-processing correction of the in-plane displacements due to their distortion resulting from the out-of-plane displacements or the shape of the specimen. The adopted experimental set-up is simple but requires precise alignment and calibration to obtain the fringe constant and other parameters required for the in-plane correction [40]. Additionally, images to be processed employing FP require a clear fringe pattern to operate adequately. On the other hand, DIC requires a speckle pattern free from periodic patterns, such as fringes, in order to allow proper correlation. Dynamic testing has attracted a great deal of interest recently for different applications (i.e., impact or vibration testing), and so both patterns should represent the same time instant and, hence, each image should present both the speckle and fringe pattern simultaneously. To make this possible without interfering with both patterns, colour encoding by employing an RGB camera is employed [37]. Particularly, a specimen should present a white background (similar to typical 2D or 3D-DIC testing) but, in this case, a red speckle is sprayed. Additionally, a white and blue fringe pattern is obliquely projected. RGB cameras with a single sensor (CCD or CMOS) record one image which presents a Bayer colour pattern [41], providing three different interpolated images, one sensitive to red, another to green and the other to blue. This methodology allows us to obtain the fringe pattern in the red image and the speckle pattern in the blue one. However, this separation is not perfect, and some residual noise could be present in images. Finally, the image processing software required for the combination of FP and 2D-DIC is straightforward and implements very efficient and simple image processing algorithms available in the literature [7,42]. The result is a simple 3D measurement system with a spatial resolution comparable to commercial 3D-DIC. Actually, this methodology has been successfully applied to different static or dynamic tests of different size of specimens [39,43,44].

However, as it was remarked, RGB pattern encoding potentially presents some aspects to be improved. Recently, Mares et al. [45] analysed several combinations of colour using FP and DIC, employing the correction algorithm developed by authors of this work [39]. In this case, the speckle pattern was not sprayed over the specimen and the proper texture of the specimen was intelligently employed as a speckle pattern to be processed with DIC algorithms. One of their main conclusions is that the best performance is gained by employing red and blue colours: specifically, blue fringes and a red light background. This interesting idea is only applicable to high textures (with high roughness) and for small displacements on which no important shadow appears. In addition, it also requires additional oblique illumination, reducing its applicability.

In the present paper, two different novel improvements in the integration of FP + 2D-DIC are presented and evaluated. Particularly, in the first alternative, a three-CCD sensor camera is employed instead of a Bayer colour camera (with a single sensor). This technology, through an optical prism, provides different images which are sensitive to each RGB colour, leading to more clear fringes and speckle patterns, improving the measurement accuracy. Nevertheless, some industrial applications require high-speed image capturing, but the availability of high-speed cameras with three sensors is very limited. Hence, an alternative to those cameras is additionally proposed. This second alternative consists of employing a single-sensor RGB camera but, instead of employing a traditional LCD projector, a red laser fringe pattern is projected. This novel combination offers the possibility of a clear differentiation of the fringe pattern from speckle pattern, leading to a better resolution in this last pattern and obtaining higher accuracy in the uncertainty of the measurement.

To evaluate the uncertainty improvement employing both alternatives, two set of tests are performed. In the first test, both methods are employed to analyse the rigid body motion of a vibrating specimen in order to compare them with the previous approach. After that, the focus is placed on employing laser projection compared to LCD projection using a single sensor camera, as this will be the case when high speed cameras are required. For this second case, the 3D displacements during a bending test of a flexible blade are analysed. For both sets of tests, the results show an interesting level of improvement of the uncertainty of the measurement, especially so for the case of the camera with three sensors.

## 2. Fundamentals

### 2.1. Fringe Projection

The principle that underlies fringe projection (FP) was first proposed in the mid-1980s [8,9] and is essentially based on parallel fringes projected over the specimen surface to determine the out-of-plane shape changes or surface deformation. These fringes should be obliquely projected in order to infer an in-plane displacement of their projection when the specimen actually experiences 3D displacement. The images of a flat reference stage and after surface deformation are analysed to extract a continuous map of out-of-plane displacements or shape.

**Figure 1:**
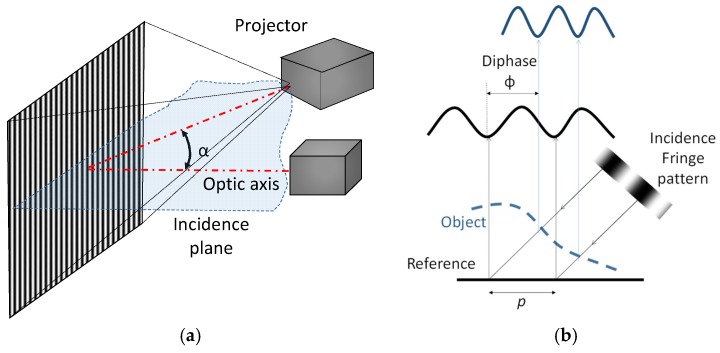
(**a**) Fringe projection schematic set-up; (**b**) illustration showing the physical principle of the fringe projection technique.

More specifically, as shown in Figure 1a, parallel straight and equidistant fringes are projected over the specimen surface with a non-zero incidence angle (α). Fringes have to be perpendicular to the incidence plane. Projected fringes are viewed by a camera placed perpendicular to the specimen surface. If the surface is completely flat (reference surface) then fringes are straight with a certain pitch value *p*. Furthermore, the intensity profile across the projected fringes has to be sinusoidal. When the specimen surface is displaced out-of-plane, then the projected fringes viewed by the camera move according to their oblique incidence angle, as illustrated in Figure 1b. Since fringes have a sinusoidal profile, their displacement can be measured as a phase shift (ϕ) which is related to the-out-of plane movement (*Z-*) as follows in Equation (1):

(1) $$z=K_{f}\phi =\frac{p}{2\pi \;\tan\alpha }\phi$$

where the *K_f_* coefficient is called the fringe constant with units (mm/rad) and depends on the pitch of the projected fringe pattern and its incidence angle.

Hence, the idea of projecting sinusoidal fringes is to encode the displacement of the fringes in the phase of the recorded fringe pattern.

The intensity profile along the *x*-direction (perpendicular to the fringes) can then be expressed as Equation (2):

(2) $$i\left(x,y\right)=r\left(x,y\right)+b\left(x,y\right)\cdot \cos\left(\frac{2\pi }{p}x+\phi \left(x,y\right)\right)$$

where *r* is the background illumination and *b* is the amplitude. Several methods exist for retrieving the phase from the fringe pattern. Those can be classified into spatial and temporal methods, with spatial methods being suitable for dynamic testing [10].

### 2.2. Digital Image Correlation

**Figure 2:**
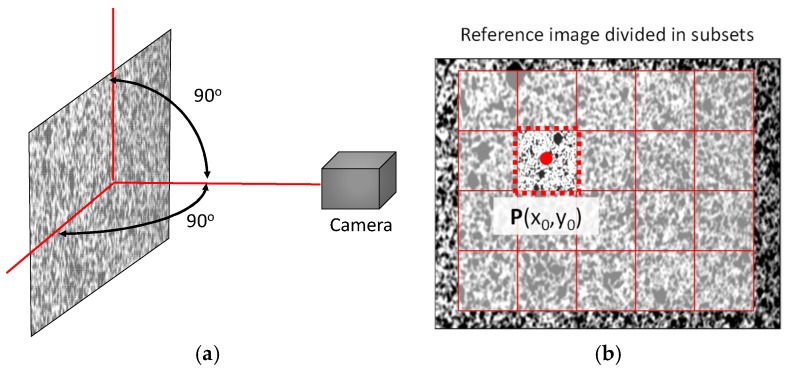
(**a**) Two-dimensional digital image correlation (2D-DIC) schematic set-up; (**b**) digital image correlation theory scheme.

Digital image correlation (DIC) is a full-field optical technique for measuring surface displacements and was first presented in the middle of the 1980s [27]. This technique is light-intensity-based and relies on the use of digital cameras and image processing capabilities. In this technique, areas of recorded images are grouped into virtual sets of pixels called facets. The illustration in Figure 2a represents the specimen surface which is divided into facets (Figure 2b) that are tracked in subsequent images when displacement occurs. Each surface element corresponding to each group of pixels from the camera is unambiguously identified by randomly painting the specimen surface with speckle, as shown in Figure 2b. Then, each surface element can be identified since the intensity pattern closed around it is matchless and will practically not change with surface deformation [16].

When a single camera is used, only in-plane displacements can be measured; this is known as 2D-DIC. A schematic representation of the 2D-DIC set-up is shown in Figure 2a. Digital images of the undeformed and deformed specimen surface are acquired during load application. Then, each facet of the image of the undeformed specimen surface is localized on the image of the deformed specimen surface by using an image correlation algorithm [42]. In-plane displacement (i.e., on the camera sensor) is obtained from the difference of the pixel position located at the centre of the facet. The real displacement of the corresponding surface element on the specimen surface is obtained by relating the pixel displacement by the lateral magnification of the used lens. When measuring in-plane displacement, it has to be considered that additional out-of-plane displacement can affect the results (if telecentric lenses are not used) since the lateral magnification will be different depending on the distance between the lens and the specimen surface [31].

The sensitivity of DIC depends primarily on the magnification of the used lens and the pixel size. Another key point of DIC is the image correlation algorithm that allows the identification and localization of the speckle images and the corresponding pixels. It is interesting to point out that digital image correlation algorithms allow the measurement of sub-pixel displacements with a resolution of about 0.1 ± 0.01 pixels [46]. For the speckle pattern, a good contrast between speckle and background is desirable. The speckle size should be big enough so that the image of the speckle is slightly bigger than the pixel size (if it is smaller, a diffuse grey pattern is observed with low contrast, and inaccurate results would be obtained).

### 2.3. In-Plane Displacement Correction

**Figure 3:**
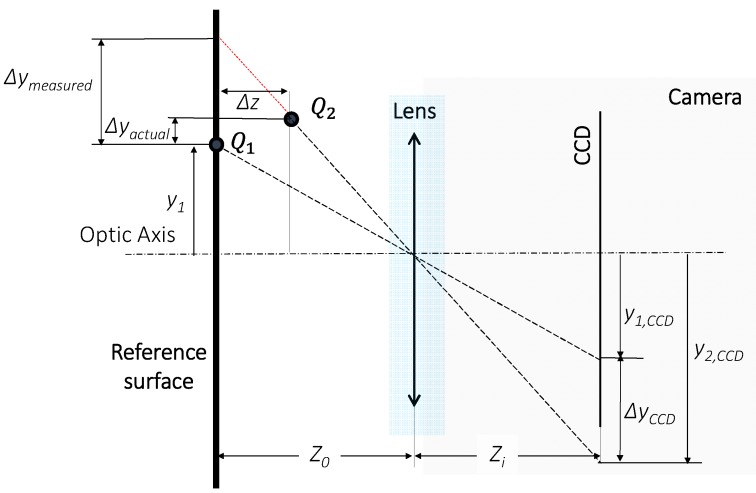
Schematic illustration showing the in-plane error induced in 2D-DIC when the specimen experiences out-of-plane displacements during a deformation process from the *Q*_1_ to *Q*_2_ position.

As previously mentioned, the in-plane displacements (Δ*x* and Δ*y*) are distorted by shape or z-displacements (Δ*z*) and they must be corrected using the out-of-plane information. The correction method adopted in the present work [39] obtains real in-plane displacement using the out-of-plane displacement obtained with FP. This correction is formulated in the base of a pin-hole model (Figure 3) with the assumption that the relative small out-of-plane displacement does not affect the image quality due to the defocusing effect.

This method associates for each pixel the corrected in-plane displacements (Δ*x* and Δ*y*) with the uncorrected ones (Δ*x_CCD_*, Δ*y_CCD_*), which are the directly obtained with 2D-DIC and the out-of-plane distance (Δ*z*) measured with FP, as illustrated in Equation (3):

(3) $$\left\{ \begin{array}{l} \Delta x=L\left[\Delta x_{CCD}-\left(x_{2,CCD}\frac{\Delta z_{2}}{\Delta z_{0}}-x_{1,CCD}\frac{\Delta z_{1}}{\Delta z_{0}}\right)\right]\\ \Delta y=L\left[\Delta y_{CCD}-\left(y_{2,CCD}\frac{\Delta z_{2}}{\Delta z_{0}}-y_{1,CCD}\frac{\Delta z_{1}}{\Delta z_{0}}\right)\right] \end{array}\right.$$

where $\left(x_{1,CCD},y_{1,CCD}\right)$ and $\left(x_{2,CCD},y_{2,CCD}\right)$ are the initial and final position of a displaced pixel at the element surface, Δ*z*_1_ and Δ*z*_2_ are the corresponding out-of-plane displacement at the corresponding pixel, *z*_0_ is the distance between the reference surface and the optical centre of the camera lens and *L* is the inverse of the lateral magnification at *z*_0_.

To perform the correction of Equation (3), some parameters are required: i.e., the distance *z*_0_, the relation *L* (mm/pixel), and the intersection of the optical axis with the CCD plane (i.e., the centre of the CCD) from which the pixel positions are referenced. In addition, FP requires the fringe constant, *K_f_* (mm/radian) and inevitably Δ*ϕ*, produced by the fringe displacement due to the *z*-displacement. All these parameters can be obtained following a calibration process [40,47].

## 3. Experimental Procedure

### 3.1. RGB Colour Pattern Encoding

As presented, to implement digital image correlation, the specimen surface has to be previously prepared by spraying it with a random speckle pattern. In addition, for fringe projection, vertical fringes have to be projected over the specimen surface. Thus, when the surface is deformed, both speckle and projected fringes moved accordingly. Two-dimensional DIC computes speckle displacements in a plane perpendicular to the optical axis while FP computes the surface displacement along the optical axis from the lateral shifting of the projected fringes. Therefore, when FP and 2D-DIC are performed at the same time, both patterns have to be conveniently separated to integrate both techniques simultaneously.

**Figure 4:**
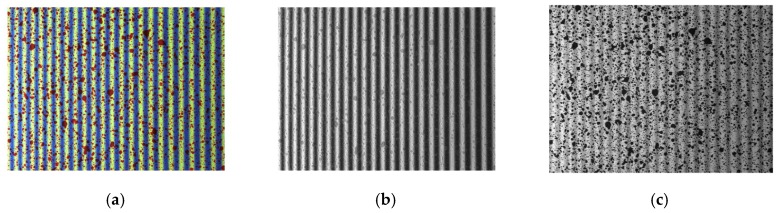
Original image RGB decomposing process. (**a**) Original RGB image; (**b**) fringe image; (**c**) speckle image.

The adopted method for the simultaneous measurement of in- and out-of plane displacements and shapes employs the projection of colour fringes and a colour (i.e., RGB) digital camera [37]. In previous approaches, the specimen was painted with red speckle over white background and blue–white fringes were projected with a sinusoidal intensity profile. According to this procedure, a single image is acquired at each loading state, as illustrated in Figure 4a. Using the Bayer filter implemented in the colour camera, speckle and fringes are then separated in two different images based on their colour: fringe images without speckle are obtained through the R-channel (see Figure 4b) and the speckle images without fringes are obtained through the B-channel (see Figure 4c). This procedure obtained appropriate results in previous applications, but in this work, two different improvements are presented and evaluated.

As commented, the main basis of the colour decoding is the manner in which RGB cameras employ a Bayer filter to obtain three interpolated images from an original raw image. This interpolation could also make the differentiation of colour more difficult. A Bayer filter consists of dividing the sensor of the camera into groups of four pixels. From them, one pixel only records the red colour, another one records blue, and finally, two pixels record the green colour. Hence, colour cameras present a lower resolution since an interpolation is required to reconstruct a full-size colour image. Only ¼ of the pixels are employed to reconstruct red (i.e., fringe pattern) and another ¼ of the pixels reconstruct a blue image (i.e., speckle pattern). The previous RGB pattern-encoding procedure neglects ½ of the total number of pixels of the sensor, and therefore a lower resolution is obtained from the images.

In this work, this drawback is overcome, more pixel information is employed and the improvement is evaluated.

#### 3.1.1. Three-Sensor Camera

**Figure 5:**
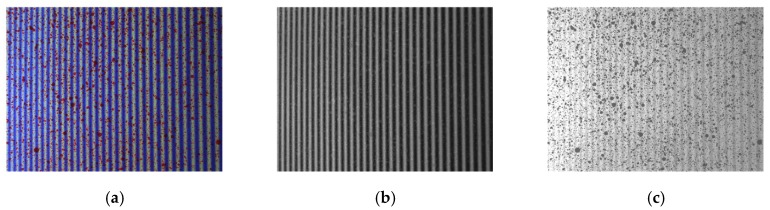
Original image RGB decomposing process. (**a**) Original RGB image; (**b**) fringe image; (**c**) speckle image.

The first proposed alternative is the employment of a camera with a beam splitter and three sensors (in this case, CCD sensors). Each of the sensors present a colour filter in order to allow each sensor to be sensitive to one specific colour: i.e., red, blue and green. Thus, the camera makes no interpolation, which facilitates better colour separation and obtains full resolution images. Hence, an image with the same red speckle and blue fringes (Figure 5a) is decomposed into a clearer fringe pattern (Figure 5b) and speckle pattern (Figure 5c) which also present a higher resolution.

#### 3.1.2. Laser Projection

**Figure 6:**
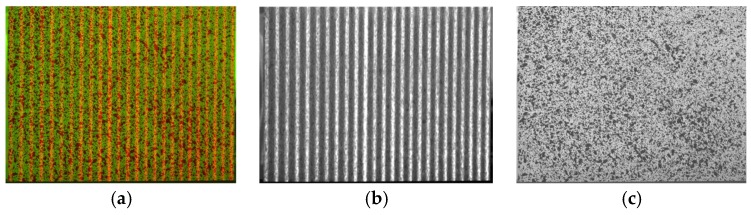
Image RGB decomposing employing the laser projection process. (**a**) Original RGB image; (**b**) fringe image; (**c**) speckle image.

The second approach attempted to obtain better resolution in speckle patterns when employing the green images obtained from single sensor RGB cameras, which represent twice the resolution employed for red or blue. For that purpose, it is necessary to project a fringe pattern with a narrow wavelength colour, far from the sensitivity of the camera to green wavelength, so that the fringe patterns do not appear in green images. Hence, a red laser fringe pattern (a laser is employed as a structured light source) was employed (Figure 6a). With this laser fringe pattern, the speckle pattern is observed with better resolution (twice the resolution compared to original approach) and with no remnants from fringes (as observed in Figure 6c). Additionally, fringes are observed in the red channel (Figure 6b) of the camera. This combination could be more appropriate when performing a high-speed test since single sensor cameras are more common and laser illumination can be free from flickering.

### 3.2. Experimental Set-Up

In this work, two different tests have been performed for the evaluation of the proposed improvement approaches, namely a vibration and a bending test. For both tests, speckle images were processed employing a commercial digital image correlation algorithm (VIC 2D from Correlated Solutions Inc., Irmo, SC, USA) and fringe patterns were processed employing Fourier transform profilometry [8]. Subsequently, each experimental set-up is explained separately.

#### 3.2.1. Vibration Test

A first test consisted of measuring in-plane (Δ*x*, Δ*y*) and out-of-plane (Δ*z*) displacements during the vibration of a rigid composite specimen. The aim was to quantify the improvements of the multisensor and laser-projection alternatives. In this test, displacements measured using single sensor (one-sensor) and multi-sensor (three-sensor) cameras are compared when employing both traditional LCD fringe projection and laser fringe projection. The three-sensor camera employed was JAI AT-200 GE (JAI Ltd., Yokohama, Japan) with 1624 × 1236 resolution pixels employing 25 mm focal length lenses. The one-sensor camera was Allied Stingray F080 (Allied Vision, Exton, PA, USA) with 1032 × 776 pixel resolution and 25 mm focal length lenses. Both cameras were arranged together and acquired images synchronously. Capturing was performed stroboscopically in order to not require a high acquisition frame rate. The optical set–up was adjusted to obtain as similar optical parameters as possible. Hence, the *L* (inverse of lateral magnification) obtained was 0.1103 mm/pixel and 0.097 mm/pixel for the one-sensor and three-sensor camera, respectively. Fringe projection was performed with one LCD Epson EB W32 (Seiko Epson Corporation, Suwa, Japan) projector (for traditional fringe projection) and one Stingray Laser (Coherent Inc., Santa Clara, CA, USA) of 660 nm wavelength and 50 mW for the new proposal where structured light is projected. The *K_f_* obtained for both systems were 0.88 mm/rad and 0.94 mm/rad for the projector and laser, respectively. The specimen was a 1 cm thick methacrylate plate of 400 mm × 250 mm sprayed with white paint and speckled with red paint (RAL code 3020).

**Figure 7:**
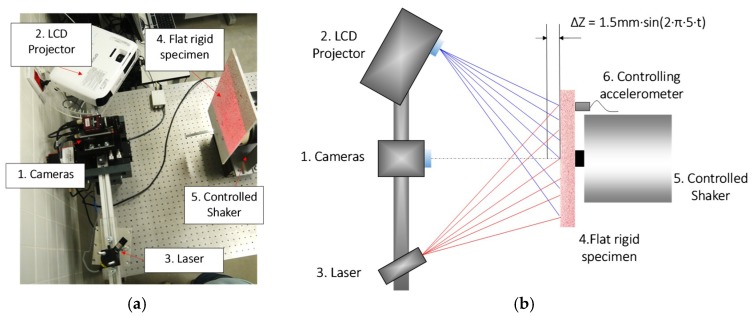
Set-up employed in vibration test. (**a**) Actual set-up; (**b**) schematic distribution.

The dynamic tests consisted of exciting the plate harmonically at a relatively low frequency, 5 Hz, in order to avoid natural frequencies of the specimen and, hence, deformation. It was screwed into the armature of an electrodynamic shaker, model GW-V20/PA30E (Data Physics Corporation, San Jose, CA, USA). The shaker and the plate were positioned while allowing that the main motion was out-of-plane from the cameras point of view, as shown in Figure 7. On the surface hidden from the cameras a tri-axial accelerometer Bruel&Kjaer 4520-001 (Bruel and Kjaer Sound & Vibration Measurement A/S, Nærum, Denmark) was fixed, as seen in Figure 7b, to monitor the plate motion. The accelerometer was employed for the close-loop control of the shaker excitation to generate a 3 mm pk–pk amplitude using a Spider 80X machine condition monitoring system (Crystal Instruments, Santa Clara, CA, USA). The excitation frequency was low enough to neglect the plate deformation due to the inertia effect. Hence, it is assumed that the amplitude was constant over the whole surface.

#### 3.2.2. Bending Test

**Figure 8:**
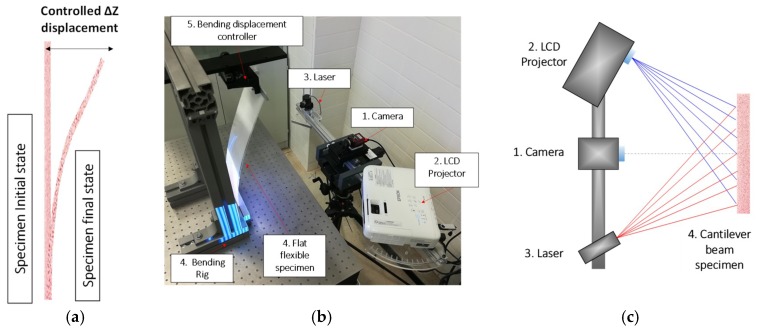
Set-up employed in bending test. (**a**) Schematic displacement inferred to cantilever beam; (**b**) actual set-up; (**c**) schematic distribution.

The second test was focused on evaluating the improvement when no multi-sensor camera is available (for instance, at high speed rates) and laser projection is used during a 3D deformation. For that purpose, controlled in-plane displacements were also required. A bending test was performed, where large Δ*y* displacement were inferred due to large Δ*z* displacement, as presented in Figure 8a. The specimen was a 500 mm × 125 mm × 2 mm polycarbonate beam rigidly clamped on its lower area, as observed in Figure 8b. The displacement to infer the bending movement was performed and micrometrically controlled at its tip as presented in Figure 8a. In this case, the same LCD projector and laser projector were employed together with the RGB camera previously described and a 25 mm focal length lens. This optical set-up resulted in a *K_f_* of 1.12 and 1.08 mm/radian for the case of laser and LCD projection and an *L* parameter of 0.1351 mm/pixel.

## 4. Results

### 4.1. Vibration Test

**Figure 9:**
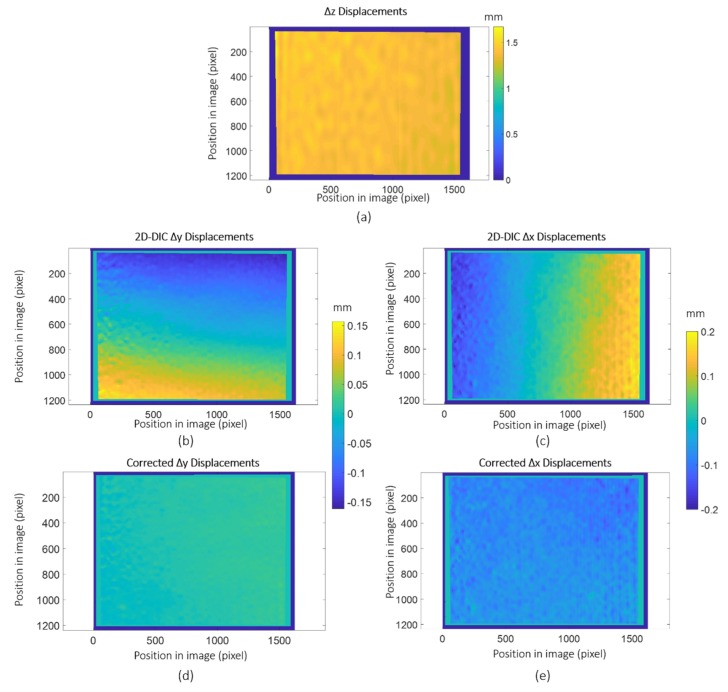
Displacement maps measured with LCD projection and a 3-CCD camera at maximum Δ*z* displacement of 1.5 mm. (**a**) Δ*z* displacement map (**b**), (**c**) Δ*x* and Δ*y* displacement map measured by 2D-DIC and (**d**), (**e**) Δ*x* and Δ*y* corrected displacement map (field of view size 157.5 mm × 119.9 mm).

For illustrative purposes, Figure 9 illustrates the displacement maps measured with LCD projection and 3 CDD camera during the vibration test at the maximum amplitude instant (Figure 9a). As observed, in-plane displacement maps (Δ*x* and Δ*y* in Figure 9b,c respectively) measured employing 2D-DIC show some aberration due to out-of-plane displacement. Those displacement are conveniently corrected employing expression (3) to obtain uniform displacement maps, as is presented in Figure 9d,e.

The correlation parameters of speckle images depend on the optical arrangement and the presence of a remnant fringe pattern on the speckle. In the case of a 3-sensor camera, speckle images were processed employing a facet size of 35 for the case of LCD and laser projections for both cases (no important remnant fringes were observed on the speckle pattern). Moreover, in the case of the 1-sensor camera, facet sizes were 41 and 35 pixels for the case of LCD and laser projections, respectively (some remnant fringes appeared on speckle pattern when employing LCD). In all cases, the step size was 1 pixel.

**Figure 10:**
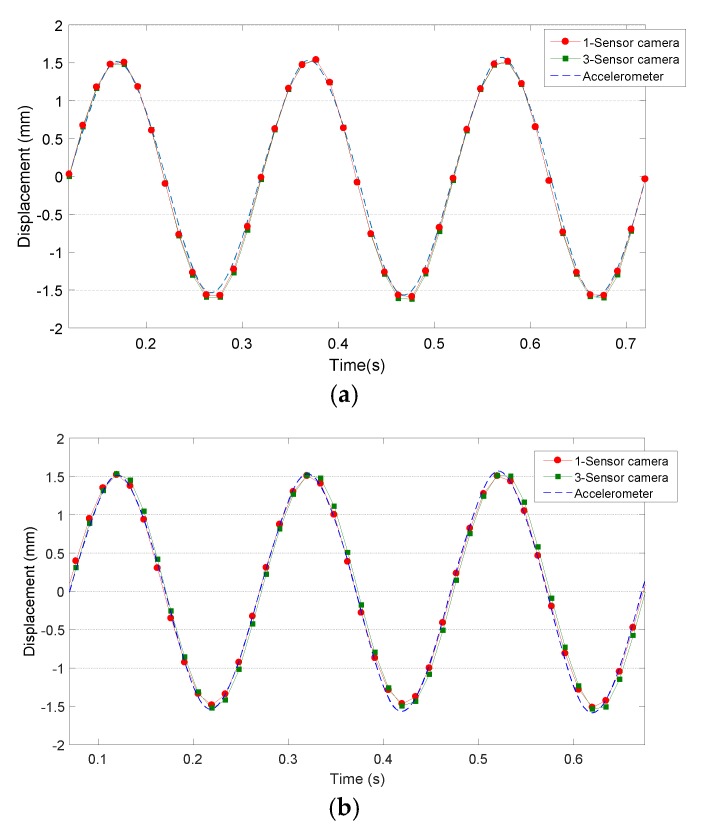
Mean Δ*z* displacement measured by 3-sensor camera (green) and 1-sensor camera (red) during vibration test employing (**a**) an LCD projector and (**b**) a laser projector together with accelerometer data (blue).

In Figure 10, the mean displacements measured on the surface of the specimen are presented together with the measurement of the accelerometer during the sine wave movement of 3 mm amplitude (1.5 mm peak). It can be detected that measurements of 3-sensor (green) and 1-sensor (red) cameras obtain similar results to those obtained by accelerometers (blue) in the case of employing an LCD projector (Figure 10a) or employing the laser projector (Figure 10b). Some differences could be observed in Figure 10a and are quantified as 0.073 and 0.051 mm for the case of the 1-sensor camera and 3-sensor camera, respectively. In the case of employing laser projection, the differences are 0.08 and 0.056 mm for the same cameras, which are small compared to the displacement amplitude of 3 mm.

In order to deeply analyze and evaluate those results, and taking into account that the specimen is not deformed but only solid rigid displacements occur, the uncertainties of the measurements are based on the calculation of the standard deviation of the mean of the full field measurements. In addition, to make a better comparison and dissociate from the small differences in resolution between different cameras and projection systems, the comparison is performed based on pixels and radians by taking into account the respective fringe order (*K_f_*) and the inverse of lateral magnification (*L*).

**Figure 11:**
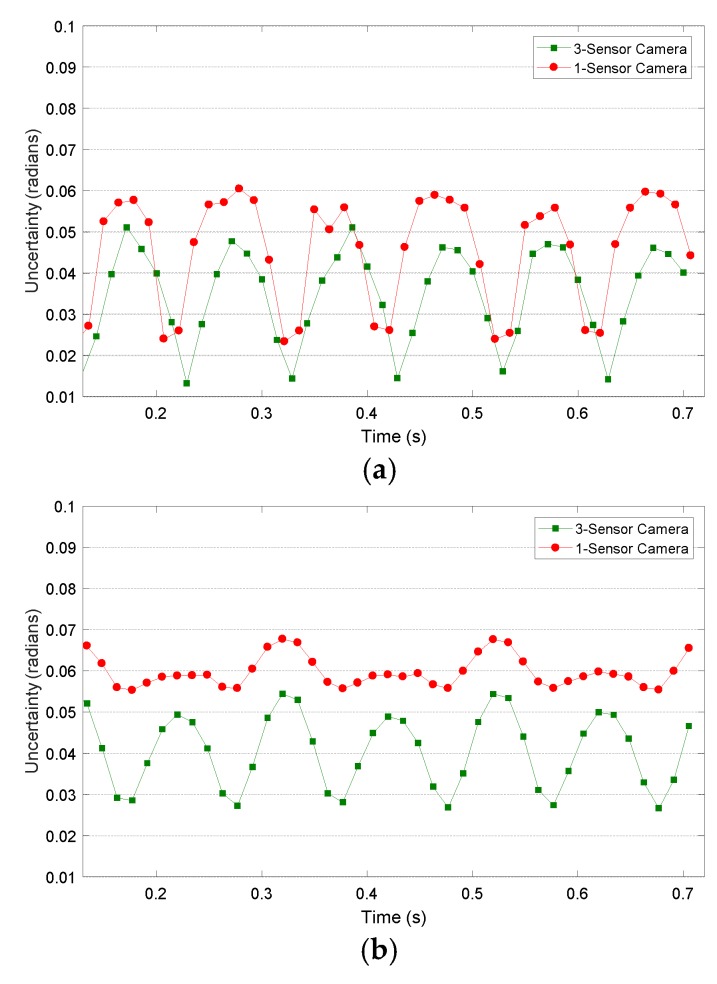
Standard deviation in Δ*z* displacement maps measured by 3-sensor camera (green) and 1-sensor camera (red) during vibration testing employing (**a**) an LCD projector and (**b**) a laser projector.

In Figure 11, the standard deviation in Δ*z* displacement maps is presented. As is observed, good results are obtained (in millimetres, representing below 1.8% of the range of displacements) and, logically, bigger standard deviations are achieved when larger Δ*z* are measured. Additionally, it is observed that the 3-sensor camera always obtains lower, and better, results, either with LCD or laser projection (Figure 11a,b respectively). It is also observed that the laser projection system always obtain more uncertainty than LCD projection (up to a 16% increase compared to the case of 1-sensor camera). Nonetheless, it is observed in Figure 11b that the value of uncertainty is more constant than in the case of LCD projection (especially so in the case of a 1-sensor camera). That shows that uncertainty is especially affected by high frequency noise inherent to laser illumination, which could be reduced by employing different filtering methodologies.

**Figure 12:**
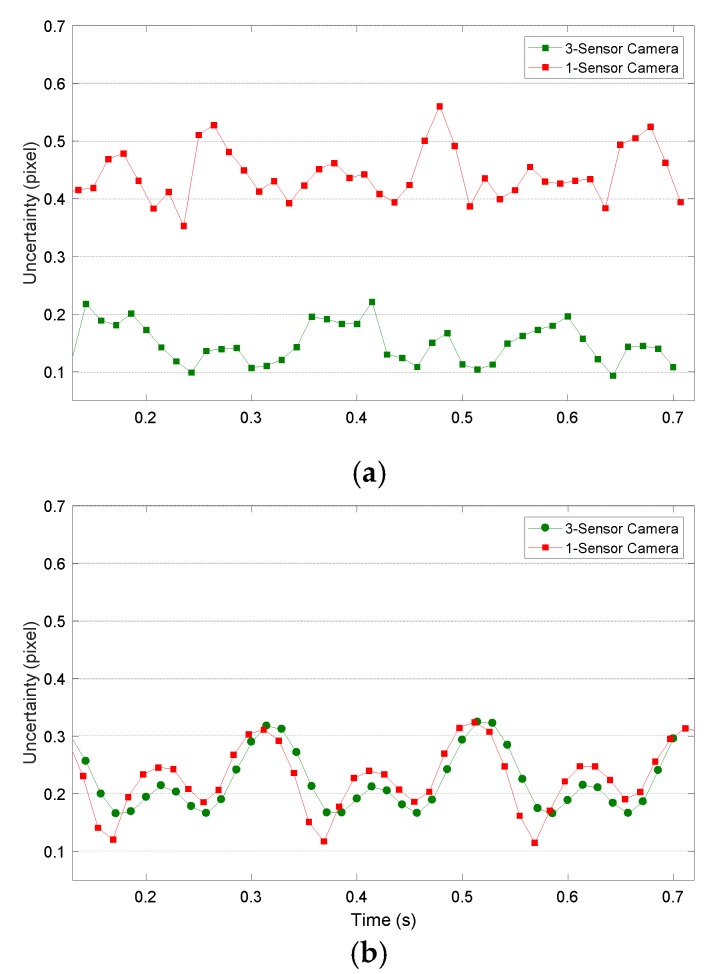
Standard deviation for in-plane displacement maps measured by 3-sensor camera (green) and 1-sensor camera (red) during vibration testing employing (**a**) an LCD projector and (**b**) a laser projector.

In Figure 12, the uncertainty of the in-plane displacements calculated as the vector magnitude of Δ*x* and Δ*y* uncertainties is presented. As happened with the uncertainty of Δ*z*, good results are obtained (always below 0.55 pixels); however, it is especially significant that the uncertainty is drastically decreased by employing either laser projection or the 3-sensor camera. This reduction could be up to 60% in the case of employing a 3-sensor camera with LCD projection (Figure 12a) or 40% in the case of laser projection, even when employing the same 1-sensor camera. This shows that interpolations inherent to the Bayer filter of cameras mainly affect speckle patterns.

### 4.2. Bending Test

As previously indicated, most high-speed cameras include only one sensor. With this limitation, as it is practically not possible to perform tests at high speed with 3-sensor cameras, the employment of laser fringe projection with a single sensor camera approach when in- and out-of-plane displacement occurs was evaluated. In fact, the bending test performed allows for a deeper analysis of the laser projection proposal when not only large Δ*z* displacement occurs but also Δ*y* displacements take place. In this case, an image when the specimen is completely unloaded was captured as a reference, and then a displacement of 130 mm was applied at the tip of the cantilever placed at 413 mm from the gripped area. The camera was focused on the area close to the clamp observing up to 96.7 mm from the grip of the specimen in order to observe the area of interest where displacement varies from negligible to large displacement.

Speckle images were processed employing a facet of 27 pixels and 35 pixels for the case of laser and LCD projection, respectively. As previously stated, this difference was required since speckle images employing LCD presenting noise from fringe patterns and bigger facets were required. It is important to highlight that a smaller facet will represent a more realistic and accurate sampling of the displacements occurring at the surface of the specimen.

**Figure 13:**
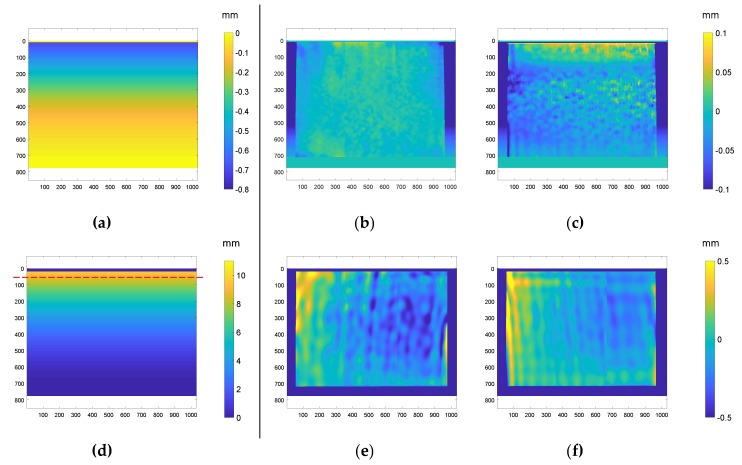
(**a**) Theroretical Δ*y* diplacement map and differences with displacement measured employing (**b**) laser and (**c**) LCD projection. (**d**) Theoretical Δ*z* displacement map and differences with displacement measured employing (**e**) laser and (**f**) LCD projection (field of view 139.4 mm × 104.8 mm).

In Figure 13, the theoretical displacement maps in the *y*- (a) and *z*- (d) direction are presented. Additionally, the differences between the theoretical and measured map employing laser or LCD projection are presented. It is observed that, for the case of Δ*y* displacements, differences for the case of LCD (Figure 13c) are noisier than those obtained with laser projection (Figure 13b). In fact, some bigger differences up to 0.1 mm are found, which represents an error of 0.74 pixels compared to the maximum error when employing a laser of 0.44 pixels. In the case of Δ*z* displacements, differences between theoretical and experiments employing lasers are slightly bigger, as expected from previous test. The maximum error employing the laser was found to be 0.47 mm (which represents a 4.95% of the maximum measured) which, taking into account the *K_f_* factor, means a difference of 0.41 radians (6.5% of the wavelength of the projected fringe pattern).

**Figure 14:**
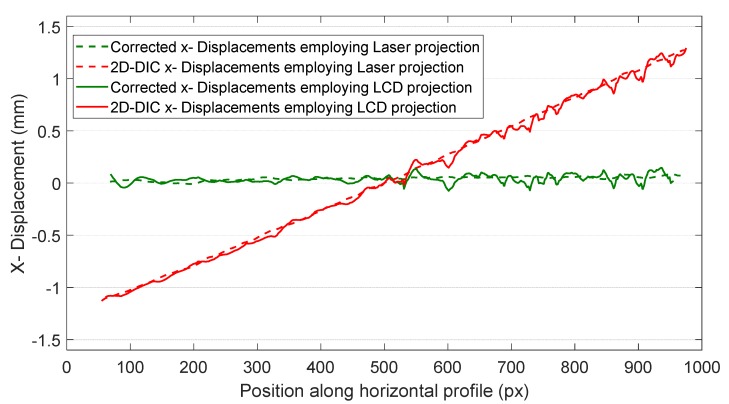
Δ*x* displacents meaured with 2D-DIC (red) and 2D-DIC corrected with fringe projection (FP) (green) along the red dashed profile in Figure 13.

In order to illustrate the behaviour of Δ*x* displacement, the measured Δ*x* displacements along a horizontal profile where a large Δ*z* occurred is presented in Figure 14 (along red dashed line in Figure 13d). As observed in the graph, displacements measured by 2D-DIC (red), which should be zero, are clearly distorted either for the case of employing laser projection (dashed line) or LCD projection (continuous line). Nonetheless, by employing expression (3), the results are adequately fitted to the actual displacement. Additionally, it is observed that results employing LCD projection are much noisier than those obtained employing laser projection (dashed line).

To summarise the results, Table 1 shows the mean differences of the theoretical and experimental displacements together with the standard deviation over the full-field displacement. It is observed that results are consistent with those obtained in the vibration test. Δ*z* displacements are slightly improved when employing LCD projection. However, displacements Δ*x* and Δ*y* are sensitively improved and uncertainty was reduced up to 40% of the results when laser projection was employed.

As observed, laser projection offers an interesting improvement for in-plane displacements. However, out-of-plane displacement could be slightly affected due to the naturally noisy nature of laser illumination. Thus, this is especially interesting when high accuracy is required for in-plane displacement, as could be the case for the calculation of strains. Strain maps could be calculated by accomplishing some strain tensors which are based on spatial derivative. Due to the nature of derivation, noise could clearly affect the final results. It is important to highlight that no filtering in the processing of images has been employed. Hence, filtering laser fringe patterns could be a point on which continue working. Moreover, an additional improvement of the measurement in Δ*z* could be also performed by decreasing the value of the *K_f_* factor, which could be simply performed by increasing the angle of the projection or increasing the pitch of the fringe pattern. Both alternatives would improve the results for out-of-plane displacements. However, in this work, this improvement has not been implemented since it was intended to have the same optical set-up to make a fair comparison between both alternatives.

## 5. Conclusions

This work presents and evaluates two improvements in the experimental methodology used for 3D displacement measurement performing fringe projection together with 2D-digital image correlation. Both alternatives improve the accuracy and uncertainty of the original experimental procedure. Among the two alternatives, it has been demonstrated that better results are obtained when employing a 3-sensor camera. Nonetheless, this kind of camera usually represents an important economic cost compared to single sensor cameras. In fact, they are not available for high-speed testing, which represents one of the most interesting fields of research in experimental mechanics nowadays. In those cases where multi-sensor cameras are not suitable, an interesting alternative has been presented by projecting a laser fringe pattern. For this case, in-plane displacements are unquestionably improved. Thus, laser projection could significantly improve the results. It has also been demonstrated that laser projection also entails some increase in the uncertainty of the Δ*z* displacement. However, it is important to highlight that, in this case, no filtering has been performed on the images.

## Figures and Tables

**Table 1:** Mean and standard deviation obtained for the LCD projection and laser projection system during bending test.

LCD Projection			Laser Projection		
Displacement	Mean Error (mm)	Standard Deviation (mm)	Displacement	Mean Error (mm)	Standard Deviation (mm)
Δ*x*	0.0268	0.0112 (0.083 px)	Δ*x*	0.0148	0.0055 (0.041 px)
Δ*y*	0.0218	0.0164 (0.1214 px)	Δ*y*	0.0084	0.0064 (0.047 px)
Δ*z*	0.07	0.0071 (0.058 rad)	Δ*z*	0.11	0.0084 (0.067 rad)
